# A feasibility study of intrafractional tumor motion estimation based on 4D‐CBCT using diaphragm as surrogate

**DOI:** 10.1002/acm2.12410

**Published:** 2018-07-03

**Authors:** Dingyi Zhou, Hong Quan, Di Yan, Shupeng Chen, An Qin, Carl Stanhope, Martin Lachaine, Jian Liang

**Affiliations:** ^1^ Key Laboratory of Artificial Micro‐ & Nano‐ structures of Ministry of Education and Center for Electronic Microscopy School of Physics and Technology Wuhan University Wuhan China; ^2^ Department of Radiation Oncology Beaumont Health System Royal Oak MI USA; ^3^ Elekta Ltd Montreal QC Canada

**Keywords:** 4D‐CBCT, diaphragm, intrafraction, motion management, SBRT

## Abstract

**Purpose:**

To investigate the intrafractional stability of the motion relationship between the diaphragm and tumor, as well as the feasibility of using diaphragm motion to estimate lung tumor motion.

**Methods:**

Eighty‐five paired (pre and posttreatment) daily 4D‐CBCT images were obtained from 20 lung cancer patients who underwent SBRT. Bony registration was performed between the pre‐ and post‐CBCT images to exclude patient body movement. The end‐exhalation phase image of the pre‐CBCT image was selected as the reference image. Tumor positions were obtained for each phase image using contour‐based translational alignments. Diaphragm positions were obtained by translational alignment of its apex position. A linear intrafraction model was constructed using regression analysis performed between the diaphragm and tumor positions manifested on the pretreatment 4D‐CBCT images. By applying this model to posttreatment 4D‐CBCT images, the tumor positions were estimated from posttreatment 4D‐CBCT diaphragm positions and compared with measured values. A receiver operating characteristic (ROC) test was performed to determine a suitable indicator for predicting the estimate accuracy of the linear model.

**Results:**

Using the linear model, per‐phase position, mean position, and excursion estimation errors were 1.12 ± 0.99 mm, 0.97 ± 0.88 mm, and 0.79 ± 0.67 mm, respectively. Intrafractional per‐phase tumor position estimation error, mean position error, and excursion error were within 3 mm 95%, 96%, and 99% of the time, respectively. The residual sum of squares (RSS) determined from pretreatment images achieved the largest prediction power for the tumor position estimation error (discrepancy < 3 mm) with an Area Under ROC Curve (AUC) of 0.92 (*P* < 0.05).

**Conclusion:**

Utilizing the relationship between diaphragm and tumor positions on the pretreatment 4D‐CBCT image, intrafractional tumor positions were estimated from intrafractional diaphragm positions. The estimation accuracy can be predicted using the RSS obtained from the pretreatment 4D‐CBCT image.

## 1 INTRODUCTION

Stereotactic body radiation therapy (SBRT) has undergone significant advancement in the past two decades. In addition to being able to deliver higher target dose while minimizing dose to the surrounding tissues and organs, SBRT utilizes minimal treatment fractions and shows advantages over conventional radiation therapy.^1^ However, respiratory‐induced tumor motion uncertainty and patient positioning uncertainty negatively impacts the treatment outcome.^2^ Various motion management techniques have been developed to reduce the discrepancy between what is planned and what is actually delivered, such as breath‐hold methods, forced shallow breathing methods, respiratory gating methods and real‐time tracking methods.^3^ Real‐time tracking is one of the promising methods for respiratory motion compensation, utilizing minimal margins and a full duty cycle.^4^

Lung tumor tracking techniques roughly fall into three categories: external surrogate tracking, internal surrogate tracking, and markerless tumor tracking.^5^ Infrared cameras, sensor belts and spirometers are examples of noninvasive and radiation‐free devices commonly used in motion monitoring systems to obtain the tracking signal.^6^ However, the correlation between external surrogate motion and internal tumor motion is not always reproducible and may vary inter and intrafractionally.^7^ Subsequently, an external surrogate alone is not accurate enough for tumor tracking, but a combination of the external surrogate with implanted fiducial markers could improve the tumor tracking accuracy.^8^ Although implanted internal surrogates provide accurate tumor position information, they also increase the risk of pneumothorax for some patients.^9^ Markerless tracking technique is an alternative method. Some studies have reported using fluoroscopic imaging for direct tumor tracking with template matching, optical flow or active shape model.^10^, ^11^, ^12^ However, direct tumor tracking would be difficult for those low‐contrast images. Previous studies have investigated the use of rotational cone beam projections for tumor tracking using template matching methods,^13^, ^14^ however, it would be difficult to ensure template matching accuracy when diaphragm or tumor was blocked by spinal cord or contralateral diaphragm at certain projection angles. Anatomic landmarks such as the diaphragm could be a surrogate for lung tumor motion.^15^, ^16^, ^17^

In this study, we hypothesized the geometrical position correlation between diaphragm and tumor remains unchanged during the same treatment fraction. Under this assumption, the spatial position of the diaphragm can be used to predict the tumor position during the posttreatment imaging or during the treatment delivery using the geometrical correlation model constructed from pretreatment CBCT images. This study mainly focused on testing this hypothesis by comparing the estimated and measured tumor position in superior‐inferior direction on posttreatment CBCT images. The tumor and diaphragm position on both pre and posttreatment CBCT images were quantitatively measured. A linear intrafraction model was constructed using tumor and diaphragm positions obtained from the pretreatment images and applied to the posttreatment CBCT images. The discrepancy (the absolute value of the difference) between the estimated and the measured tumor positions on posttreatment images was investigated. Receiver operating characteristic (ROC) analysis was performed to test the predictive power of different anatomical and model parameters on the tumor position estimation accuracy. This study will potentially guide our future study which may focus on real‐time tumor position estimation based on Cone Beam projection images.

## 2 MATERIALS AND METHODS

### 2.A Patient data

Twenty SBRT lung patients treated using three to five fractions were included in this IRB‐approved retrospective study. Table 1 summarizes the patient information and tumor characteristics. Patients were setup and treated in head‐first supine position and free‐breathing state after being immobilized with vacuum cushions. 4D CBCT images were acquired using a gantry‐mounted CBCT scanner (Symmetry, Elekta Oncology System) with slow gantry rotation speed preset. Each CBCT scan occurs over 200° of gantry rotation. A total of 85 paired (pre and posttreatment) daily 4D‐CBCT images were acquired during the treatment course. Each 4D‐CBCT image included 10 respiratory motion phases. The median time interval between pre and posttreatment 4D‐CBCT images was 19.48 (10.27–31.20) min.

**Table 1:** Summary of 20 patient information and tumor characteristics

Age	Gender	GTV (cc)	GTV Excursion (mm)	Tumor Location
Median 77	Male 50%	Median 6.87	Median 7.8	LLL 50%
Range 59–89	Female 50%	Range 0.85–48.81	Range 4.1–26.8	RLL 25%
				RML 25%

LLL, Left Lower Lobe; RML, Right Middle Lobe; RLL, Right Lower Lobe.

### 2.B Motion data of diaphragm and tumor at the treatment

For each set of patient planning 4D‐CT images, tumor and diaphragm were first delineated on a reference phase of planning 4D‐CT images and then propagated to the remaining nine phase images with necessary manual adjustment. For each CBCT phase, tumor and diaphragm ROIs were deformed from the corresponding planning 4D‐CT phase image using ADMIRE, Research version (Elekta AB, Stockholm, Sweden). Propagated contours were reviewed carefully and manually adjusted if necessary. For each pair of pre and posttreatment CBCT images, a bony registration was first performed between the average image of pre and posttreatment CBCT to exclude the effect of the rigid‐body bony movement after pretreatment imaging. Tumor and diaphragm positions were registered to the reference CBCT phase image for each of the other 19 phase images. Tumor positions were obtained using contour‐based translational alignment for each phase image. Diaphragm positions were obtained using the translational alignment of its apex position.

### 2.C Construction and evaluation of intrafraction model

**Figure 1:**
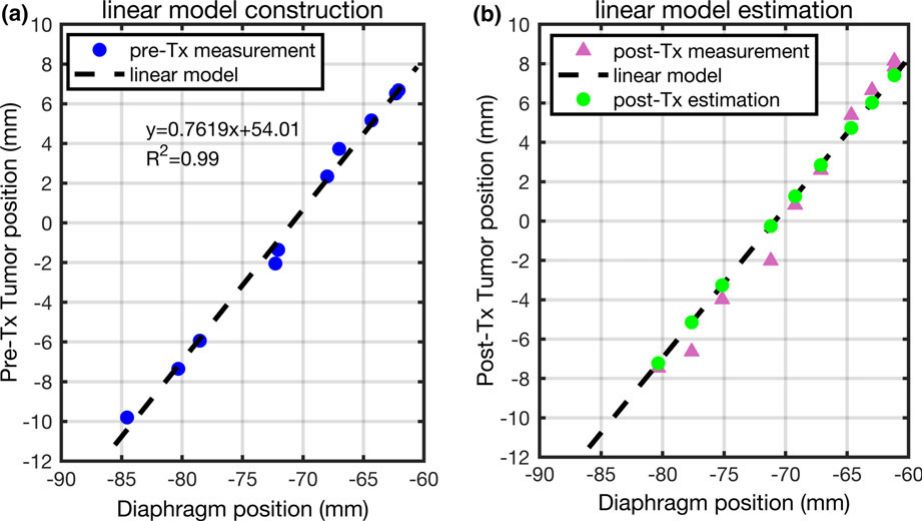
Example of (a) a linear model construction using the tumor and diaphragm positions measured on pretreatment CBCT image, and (b) measured and estimated tumor position on posttreatment CBCT.

For each patient treatment fraction, a linear intrafraction model was constructed by performing linear regression between the tumor and diaphragm positions manifested on the pretreatment 4D‐CBCT images. Using this intrafraction model, the tumor positions on posttreatment CBCT images were estimated and compared with their corresponding measured results. Figure 1 shows an example of the linear intrafraction model and the corresponding application on posttreatment images for a specific treatment fraction.

Accuracy of the linear intrafraction model was evaluated by investigating the discrepancy between the estimated and measured tumor position on posttreatment 4D‐CBCT images. Three types of error metrics were studied. There included phase estimation error, which is defined as the absolute value of the per‐phase difference in tumor position; mean position error, which is defined as the absolute value of the difference in mean tumor position over the 10 phase images; and excursion error, which is defined as the absolute value of the difference in tumor excursion.

### 2.D Accuracy test of tumor position estimation

ROC analysis was performed to test the predictive power of the patient anatomical or model parameters on the tumor motion position estimation accuracy using only the information from pretreatment CBCT images. Investigated parameters included: the diaphragm motion excursion, the tumor motion excursion, the relative distance between diaphragm and tumor in SI direction, the slope and intercept of the linear intrafraction model, and the residual sum of squares (RSS). All these parameters were obtained from the pretreatment 4D‐CBCT images and used to predict the tumor position estimation error evaluated on the posttreatment 4D‐CBCT images.

In the situation in which the tumor per‐phase estimate error was within 3 mm, the event was defined as a true positive. In contrast, error greater than 3 mm was defined as a true negative. False positives and false positives were defined as is logical. Sensitivity and specificity (also known as the true positive rate and true negative rate) represent the probabilities of using an investigated parameter to correctly identify patients with tumor position estimate error less than or larger than 3 mm. The area under the curve (AUC) was calculated using R package *verification* and the corresponding *P*‐value was calculated based on Mann–Whitney *U* test. The optimal cut‐off for a given investigated parameter was determined by maximizing the Youden index (i.e., sensitivity + specificity − 1) using R package *OptimalCutpoints*.

## 3 RESULTS

### 3.A Tumor and diaphragm intrafraction variation

**Figure 2:**
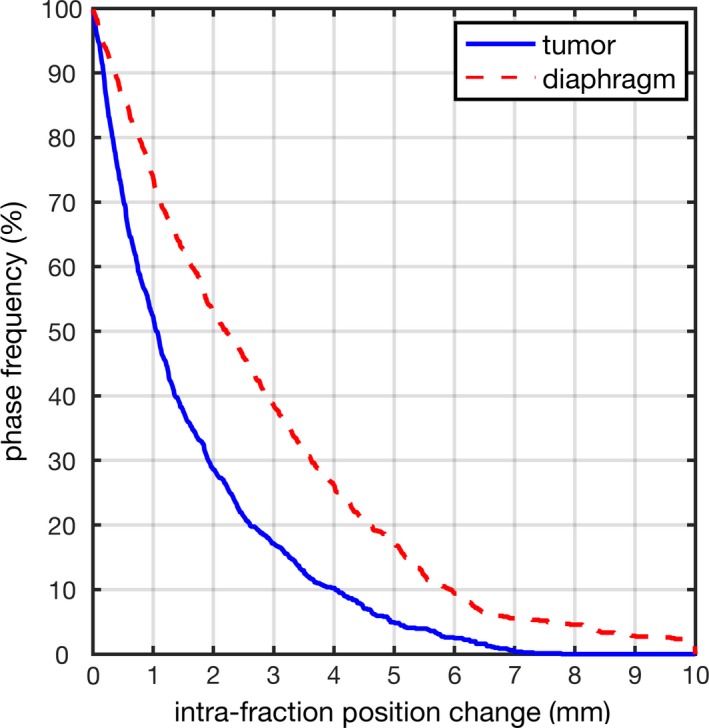
Cumulative distribution of tumor and diaphragm position change directly compared between pre and posttreatment.

Tumor and diaphragm positions were measured and directly compared for both pre and posttreatment CBCT images. The per‐phase position changes (defined as the absolute value of position difference) were 1.60 ± 1.58 mm for tumor and 2.81 ± 2.39 mm for diaphragm. Tumor mean position and excursion changes were 1.51 ± 1.51 mm and 0.99 ± 0.98 mm, respectively. Figure 2 shows the cumulative distribution of tumor and diaphragm per‐phase position change, tumor and diaphragm intrafraction positions change are greater than 3 mm in 17% and 39% of the phases.

Pearson correlation coefficients were calculated between the diaphragm and tumor positions obtained from pre and posttreatment images to evaluate the relationship stability. The mean ± SD correlation coefficients were 0.984 ± 0.017 and 0.979 ± 0.023 with respect to the pre and posttreatment images.

### 3.B Tumor position estimation accuracy

**Figure 3:**
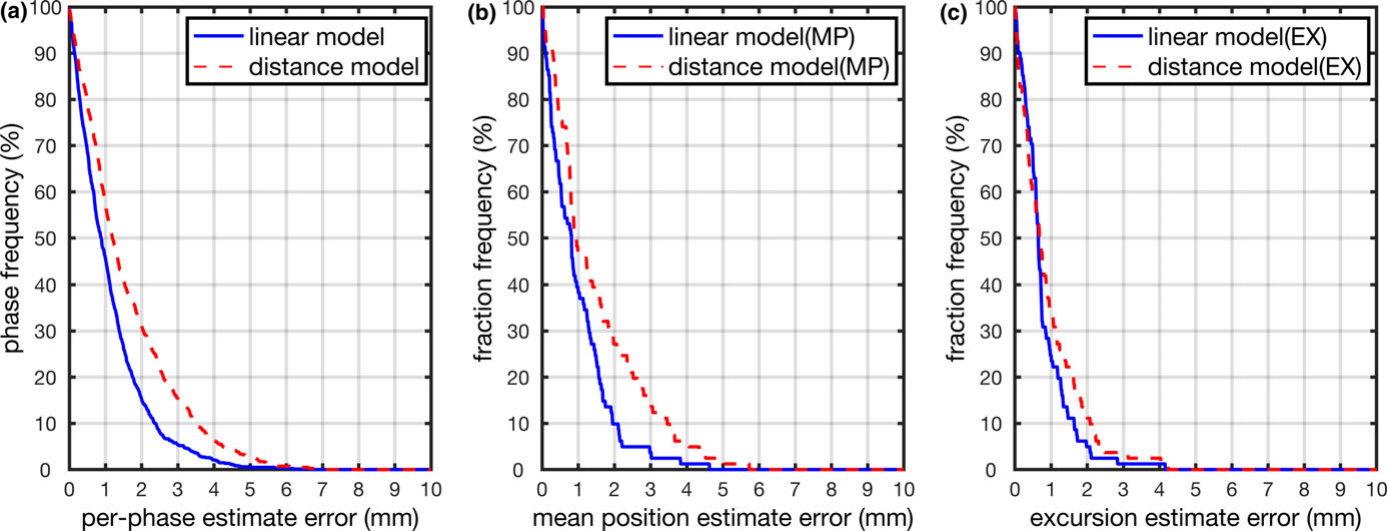
Cumulative distribution of tumor estimation error (a) per‐phase (b) mean position, and (c) respiratory motion excursion.

Tumor position estimation accuracy using the linear intrafraction model is listed in Table 2. For comparison purposes, we also investigated a distance model which assumed the per‐phase tumor positions relative to the diaphragm on the pre and posttreatment 4D‐CBCT images were unchanged. Figure 3 shows the cumulative histogram of the tumor per‐phase position, mean position, and excursion estimate errors using the linear model and distance model, respectively. For the linear intrafraction model, the per‐phase position error was within 3 mm for 95% of phases. Mean position and excursion estimate errors were within 3 mm for 96% and 99% of fractions, respectively. In comparison, when using the distance model, the errors were within 3 mm for 85%, 85%, and 96%, respectively. 3 mm uncertainty is a critical value to our current margin design.

**Table 2:** Tumor position estimation accuracy and the tumor position change between pre and posttreatment

(Mean ± SD, mm)	Per‐phase	Mean position	Excursion
Linear model	1.12 ± 0.99	0.97 ± 0.88	0.79 ± 0.67
Distance model	1.61 ± 1.34	1.50 ± 1.29	0.93 ± 0.88
Direct comparison	1.60 ± 1.58	1.51 ± 1.51	0.99 ± 0.98

**Figure 4:**
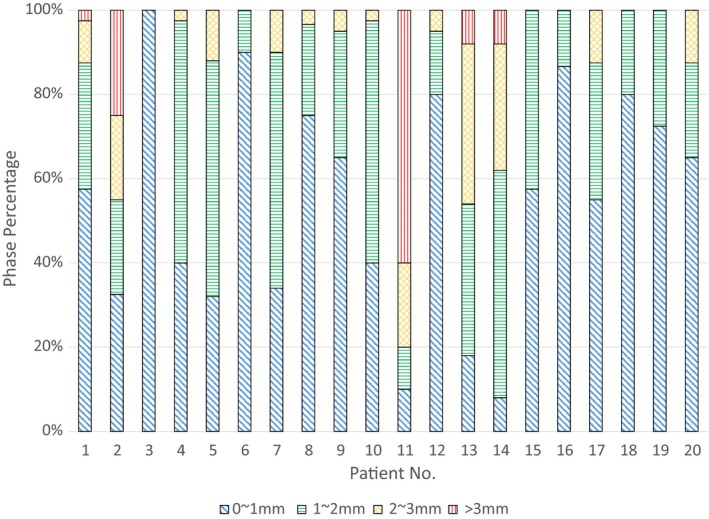
Tumor position estimate error distributions for the 20 individual patients.

The tumor position estimate error varied patient to patient. Figure 4 shows the tumor position estimate error distribution for the 20 individual patients using the linear intrafraction model. Patients No.2 and No.11 had the greatest tumor position estimation error; estimation error was greater than 3 mm for more than 25% of the phases when using the linear intrafraction model. For the other 18 patients, estimation error was greater than 3 mm for no more than 8% of phases.

### 3.C ROC analysis

Results of the ROC analysis are listed in Table 3. All Investigated parameters except diaphragm excursion had a significant predictive value (*P* < 0.05) when predicting tumor position estimate error using the linear intrafraction model. The residual sum of squares (RSS) achieved the highest predictive value with an AUC of 0.92 and a corresponding optimal cut‐off of 3.91 mm^2^. At this cut‐off point, the sensitivity and specificity were 0.77 and 0.98. This indicates that if the RSS obtained from the pretreatment 4D‐CBCT image is smaller than 3.91 mm^2^, there is a 77% probability that the tumor phase position estimate error is less than 3 mm when using the preconstructed linear intrafraction model for this treatment fraction. Otherwise, if the RSS is larger than 3.91 mm^2^, the estimate error will be larger than 3 mm 98% of the time.

**Table 3:** Predictive power of the parameters on tumor position estimate error

Parameters(unit)	Criteria (mm)	AUC	*P*‐value	Optimal cut‐off	Sensitivity	Specificity
DIA_EX (mm)	3	0.54	0.247	9.61	0.19	0.97
GTV_EX (mm)	3	0.78	0.000	12.62	0.75	0.81
L (mm)	3	0.64	0.017	70.45	0.96	0.47
Slope	3	0.79	0.000	0.75	0.57	1.00
Intercept	3	0.66	0.001	54.49	0.94	0.56
RSS (mm^2^)	3	0.92	0.000	3.91	0.77	0.98

*P*‐value was calculated based on Mann–Whitney *U* test (Null Hypothesis: AUC = 0.5).

DIA_EX, diaphragm excursion; GTV_EX, GTV excursion; L, the distance between diaphragm and tumor in superior‐inferior direction; Slope and Intercept, coefficients obtained from linear regression; RSS, residual sum of squares; AUC, area under the curve.

## 4 DISCUSSION

Intrafractional variation in lung tumor position due to respiration and baseline shift/drift during dose delivery has been discussed previously in literature.^18^, ^19^, ^20^, ^21^ From this study, the absolute differences in mean tumor position and excursion between pre and posttreatment images were found to be 1.51 ± 1.51 mm and 0.99 ± 0.98 mm, respectively. These values are in good agreement with a previous study.^20^ A strong linear correlation was observed between the diaphragm and tumor respiratory motions; mean Pearson's correlation coefficient from all 4D‐CBCT images was larger than 0.98. Therefore, a linear intrafraction model could be appropriate for modeling the relationship between diaphragm and tumor motion. To test the hypothesis that the geometrical correlation is stable, the models were applied on posttreatment images. Tumor positions were estimated from diaphragm positions and compared with actual positions.

Table 2 clearly demonstrated the superiority of the linear intrafraction model than the distance model. Distance model discrepancy was on the same order of magnitude as tumor position variation when comparing pre and posttreatment images. Significant differences were observed between linear intrafraction model and distance model (*P* < 0.05). Compared with distance model, the linear intrafraction model achieved 0.48 ± 1.11 mm improvement on per‐phase estimation accuracy, 0.52 ± 0.85 mm on mean position accuracy, and 0.14 ± 0.72 mm on excursion estimation accuracy. This indicates that the relative distance between tumor and diaphragm positions were less stable compared with their geometric correlation. With the preconstructed intrafraction model, the tumor intrafraction positions could be estimated using diaphragm positions as inputs to the model. The model could be further explored for markerless tumor tracking during dose delivery using intrafraction diaphragm motion as a surrogate. For respiratory‐gated radiotherapy, size and position of the gating window were usually determined from each patient's unique respiratory trace.^22^, ^23^ Accurate tumor mean position and excursion are essential information for defining the gating window. When using the linear model, mean position and excursion estimation errors were 0.97 ± 0.88 mm and 0.79 ± 0.67 mm. The overall good estimation accuracy on the tumor mean position and excursion demonstrates the potential of using the linear intrafraction model to define an appropriate window for tumor gating. Due to the hysteretic nature of the breathing process, a quadratic model which separates the respiratory motion into exhalation and inhalation was also investigated. However, the quadratic model was worse on tumor phase position estimate accuracy (1.21 ± 1.32 mm).

The linear intrafraction model demonstrated superior estimate accuracy. However, results in Fig. 4 demonstrated that the estimate errors were fraction specific. Therefore, being able to identify those fractions with accurate/inaccurate tumor position estimations prior to the estimation process is essential. Once posttreatment CBCT images are replaced with intrafraction images, pretreatment image parameters can be used to determine whether the intrafraction model could be stable during treatment. RSS achieved the highest predictive value in this study and thus is a best indicator for fraction selection. For instance, Patient No.2 and No.11 showed large RSS values with mean ± SD of 23.67 ± 5.04 and 12.75 ± 5.86 mm^2^, respectively. Excluding those fractions with RSS > 3.91 mm^2^, the intrafraction tumor per‐phase position, mean position, and excursion estimation errors decreased to 0.88 ± 0.64 mm, 0.77 ± 0.57 mm, and 0.78 ± 0.74 mm, respectively. Correspondingly, estimation errors were less than 3 mm 99%, 100%, and 100% of the time. Such indicators have the potential to impact clinical decision‐making. Specifically, these metrics indicate whether the linear intrafraction model constructed prior to treatment can be reliably used for subsequent intrafraction motion management.

## 5 CONCLUSION

Utilizing the relationship between diaphragm and tumor positions on the pretreatment 4D‐CBCT image, intrafractional tumor positions can be estimated from intrafractional diaphragm positions. Furthermore, the estimation accuracy can be predicted using the RSS determined from the pretreatment 4D‐CBCT image.

## CONFLICT OF INTEREST

The authors have no conflict of interest to disclose with respect to this study.
